# Reduced Graphene Oxide Derived from Low-Grade Coal for High-Performance Flexible Supercapacitors with Ultrahigh Cyclability

**DOI:** 10.3390/nano12172989

**Published:** 2022-08-29

**Authors:** Yi-Ming Wang, Chun-Hua Zhang

**Affiliations:** College of Safety Science and Engineering, Liaoning Technical University, Fuxin 123000, China

**Keywords:** reduced graphene oxide, efficient utilization of coal, cost-efficient production, flexible supercapacitor, ultrahigh cyclability

## Abstract

Preparation of reduced graphene oxide (RGO) from abundant and inexpensive low-grade coal is regarded as one of the most promising methods for utilizing this resource in a high-value and environmentally sustainable manner. As the main precursor for the fabrication of RGO, graphene oxide (GO) can be extracted from low-grade coal such as lignite, but its size is just in the range of tens to hundreds of nanometers, which limits its practical application. Herein, we demonstrate that large-size RGO sheets can be prepared in large quantities by the pretreatment of lignite using the high temperature–high pressure (HTHP) method. The RGO electrode after the reduction reaction by 50 mM NaBH_4_ at 105 °C features porosity and high conductivity, which can facilitate high electrochemical reaction efficiency. Thus, we also demonstrate the use of lignite-derived RGO for supercapacitor electrode materials with high performance. The lignite-derived RGO supercapacitor can deliver outstanding volumetric capacitance (30.6 F cm^−3^), high energy density (4.2 mW h cm^−3^), excellent flexibility (79.5% retention of the initial capacitance at 180° bending), and a long lifespan (112.3% retention of the initial capacitance after 20,000 cycles). It is believed that the proposed large-size RGO based on reasonable optimization of inferior lignite will offer a new prospect for next-generation energy storage applications.

## 1. Introduction

As epidermal and intelligent electronics become more prevalent in our daily lives, there is a high demand for flexible and high-performance energy storage devices [1,2,3]. Flexible supercapacitors are expected to become an emerging focus among many energy storage devices, owing to their outstanding characteristics, including fast charge–discharge rate, high output power density, high cyclic stability, especially in flexibility and easy integration [4,5,6]. Recently, a lot of works have been devoted to the development of flexible high-performance supercapacitors using novel carbon nanomaterials and their composites [7,8]. Among the various carbon nanomaterials, graphene is a significant type of important functional material which has attracted intensive attention for its promising applications in supercapacitor electrode materials, primarily because of its exceptional electrical conductivity, robust mechanical flexibility, notably large specific surface area, and exceptional chemical inertness to the surrounding environment [9]. Several recent studies have shown that graphene has been explored as an outstanding electrode material in symmetric supercapacitors and shows superior specific capacitance and rate performance [10,11]. However, the mass manufacturing of graphene in a low-cost way is a significant barrier to its widespread industrial application.

Over the past few years, many endeavors have been made toward the preparation of graphene. Mechanical exfoliation using the Scotch-tape method and chemical vapor deposition (CVD) can usually produce good-quality graphene, but are inappropriate for industrial manufacture on a large scale because of their low yield and high cost [12,13]. Some economical and practical honeycomb-like carbon sheet, e.g., reduced graphene oxide (RGO), characterized by plentiful functionalization beneficial to improve the electrochemical performance of flexible supercapacitors, can be regarded as a vital member of the graphene family [14]. Therefore, the formation of individual graphene sheets by chemical reduction of graphene oxide (GO) is found to be the most promising method for scalable and cost-efficient production of graphene-based materials.

Generally, GO was utilized as a precursor for producing graphene-based materials in large quantities [15]. Nevertheless, challenges still exist in the mass production and commercialization of chemically exfoliated graphene. For example, the majority of starting material utilized in this method (i.e., highly pure graphite) is high-priced (at least ten times higher than that of industrial graphite) [16]. In order to alleviate this issue, natural carbonaceous materials can be transformed to graphitic materials used for electronics, sensors, biomedicine, and energy storage and conversion [17]. Coal is a natural carbon material that can be broadly classified as either lignite, bituminous coal, or anthracite, depending on the metamorphosed extent. Among them, lignite and sub-bituminous coal are called low-grade coal due to their characteristics of having high moisture content, high ash content, high volatile matter content, and low quantitative value [18,19]. Traditionally, low-grade coal was typically converted into fuel by carrying out a variety of processes including combustion, pyrolysis, gasification, and liquefaction [20]. However, traditional utilization methods confront daunting challenges, such as inefficient energy conversion and substantial environmental pollution [21,22]. Therefore, it is necessary to develop a high-value and environmentally sustainable method for utilizing low-grade coal [23]. Compared to natural flake graphite and other precursors, coal molecules are made up of a specific number of aromatic units, as well as short aliphatic and ether links [24], so coal may be a good candidate for developing carbon nanomaterials due to its multilevel nanoarchitecture and specific functions. Savitskii et al. [25] employed a thermo-oxidative approach to create colloidal GO nanoparticle dispersions ranging in size from 122 nm to 190 nm using anthracite coal. It has been demonstrated by Pakhira et al. [26] that GO can be extracted by leaching low-grade coal with HNO_3_, but such GO sheets are more likely to fragment into small spherical shapes in a few tens of nanometers. It is well known that an increase in the degree of orientation of the crystalline structure of coal will benefit the increase of sheet size [24]. Recently, we showed that the high temperature–high pressure (HTHP) method can be used to quickly fabricate highly ordered graphite from lignite, which can be referred as an ideal precursor for graphene oxide [27].

In this study, we demonstrated the synthesis of stable GO from lignite-based synthetic graphite using the Hummers method. Furthermore, the RGO was prepared by employing the reducing agent, sodium borohydride (NaBH_4_). Using X-ray photoelectron spectroscopy (XPS) and Fourier transform infrared spectrometer (FTIR), the C:O ratio and surface information about the residual functional groups of RGO were investigated. The structure and chemical components of RGO obtained under various molar concentrations of NaBH_4_ and treatment temperatures are investigated by XRD, Raman spectroscopy, and HRTEM to trace their structural evolution during reduction reaction. Application of RGO derived from lignite as the electrode material of flexible supercapacitors demonstrated that the as-prepared electrode exhibits excellent energy storage performance along with outstanding flexibility and superior cycling stability. These significant features verify the viability of lignite-derived RGO in the application of the flexible supercapacitor.

## 2. Experimental Section

### 2.1. Synthesis of GO Powder

Bulk highly ordered graphite was synthesized from lignite (Puhe Coal Mine, Shenbei Mining Area, Liaoning Province, China) under high temperature (1300 °C) and high pressure (6 GPa) [27]. After that, GO was synthesized from the aforementioned lignite-based synthetic graphite via a conventional Hummers method [28]. Typically, lignite-based synthetic graphite powder (1.0 g) and NaNO_3_ (0.5 g) were dispersed in concentrated H_2_SO_4_ (23 mL) and placed in an ice bath environment with stirring. Under constant agitation, KMnO_4_ (3.0 g) was slowly added to prevent the reaction temperature from rising above 20 °C. Successively, the mixed suspension was transferred to a 35 °C oil bath for 1 h and the reaction temperature was further increased to 85 °C before attenuation and washing by 100 mL of deionized water and 15 mL of H_2_O_2_ (30%). The mixture was filtered and then washed with HCl aqueous solution (5%), ethyl alcohol, and deionized water in sequence until the pH value became around 7. After being purified by dialysis for one week, the GO powder was collected after freeze-drying. For comparison, GO (diameter: 0.5–5 μm, thickness: 1–3 nm) was also acquired from Nanjing XFNANO Materials Tech Co., Ltd., located in China.

### 2.2. Reduction of GO and Preparation of Electrodes

To confirm the effect of the NaBH_4_ reducing agent, 10 mg of GO powder was immersed in NaBH_4_ solutions (20 mL) with varying concentrations (15, 50, and 100 mM) at room temperature (RT) and kept for 4 h. The products were labeled as RGO-RT-15, RGO-RT-50, and RGO-RT-100 corresponding to reduction concentrations of 15, 50, and 100 mM, respectively. To deepen the reduction of GO, GO powder was also dipped in aqueous NaBH_4_ (50 mM) at 105 °C for 4 h, and the final product was referred to as RGO-105-50. For the comparison of energy storage performance of RGO from commercial GO and lignite-derived GO, the purchased GO was reduced at the same condition of RGO-105-50, and the as-prepared product was named as c-RGO-105-50. To fabricate film electrodes, the post-reduction GO dispersions were vacuum filtered through a porous alumina membrane (Whatman, 20 nm pore size, 47 mm diameter) to make a homogenous thin film, which was then vacuum-dried at 70 °C for 24 h.

### 2.3. Preparation of Supercapacitor Devices

For the three-electrode system, a platinum sheet and Ag/AgCl electrode were used as the counter electrode and reference electrode, respectively. All the electrodes were prepared by mixing 80% as-prepared RGO, 10% PVDF, and 10% carbon black in NMP to form a homogeneous slurry. After that, the slurry was homogeneously blade-coated onto carbon cloth, and the as-prepared electrodes were dried in a vacuum oven at 80 °C for 10 h. The mass of active material on the coating area (1 cm^2^) was 2–3 mg.

In the assembly of the flexible supercapacitor, the electrode film was transferred on the gold-coated PET film, before porous alumina was etched by 3 M NaOH solution. The electrodes were placed into a vacuum oven for 12 h at 60 °C before the copper tapes were adhered on one edge of each Au-coated PET piece with silver plastic. The following steps were taken to make the PVA/H_3_PO_4_ electrolyte [29]: 3 g of PVA was dispersed in 30 mL of deionized water and heated to 85 °C under continuous stirring until a clear solution was obtained. The PVA/H_3_PO_4_ electrolyte was then spread on the RGO films. After drying to get rid of any extra water, two pieces of Au-coated PETs were pressed together to create a device with a classic sandwich structure. The electrode volumes of GO, RGO-RT-15, RGO-RT-50, RGO-RT-100, RGO-105-50, and c-RGO-105-50 were determined to be around 2.05 × 10^−4^ cm^−3^, 2.15 × 10^−4^ cm^−3^, 2.4 × 10^−4^ cm^−3^, 2.15 × 10^−4^ cm^−3^, 2.25 × 10^−4^ cm^−3^, 4.85 × 10^−4^ cm^−3^, and 5.2 × 10^−4^ cm^−3^.

### 2.4. Characterization

X-ray diffraction (XRD) was carried out on X’Pert3 diffractometer with a Cu-Ka target. The field emission scanning electron microscopy (Carl Zeiss Ultra 55, Oberkochen, Germany) and transmission electron microscope (JEOL-2100PLUS, Tokyo, Japan) were used to characterize the morphology and nanostructure of the samples, respectively. Raman spectra (HORIBA LabRAM HR Evolution, Kyoto, Japan) were obtained through the use of a 532 nm laser as the light source. The XPS measurements were carried out on an Axis Ultra spectrometer (ESCALAB 250Xi, Thermo Fisher, Waltham, MA, USA), and the spectra were calibrated to the C *1s* peak, which was located at 284.5 eV. Fourier transform infrared spectrometer (Thermo Scientific Nicolet iS20, Waltham, MA, USA) was utilized in order to acquire the surface information of GO and RGO. The resistance of the RGO films was measured using the four-point probe method (Keithley 2400, Beaverton, OR, USA).

### 2.5. Electrochemical Measurements

All of the electrochemical measurements were evaluated using a CHI-660E electrochemical workstation at room temperature. These measurements included cyclic voltammetry (CV), galvanostatic charging–discharging (GCD), and electrochemical impedance spectroscopy (EIS). The CV curves were recorded using different scan rates ranging from 5 mV s^−1^ to 10 V s^−1^ in the potential range of 0.0 to 1.0 V. Different current densities of 0.03 to 0.2 A cm^−3^ were used to conduct GCD tests. EIS was used to characterize frequency-dependent behaviors in the frequency range of 100 kHz to 0.01 Hz at open circuit potential with a 5 mV ac perturbation.

For three-electrode system, the gravimetric capacitance (C_g_) calculated by galvanostatic discharge curves was obtained by the following formula [30,31].

(1) $$C_{g}=\frac{I\Delta t}{m\Delta V}$$

The specific volumetric capacitance values (C_V_, F cm^−3^) were evaluated from the CV curves by the formula [29].

(2) $$C_{V}=\frac{\int i\left(V\right)\mathrm{dV}}{2\mathrm{Vv}\Delta V}$$

The specific gravimetric capacitance values (C_g_, F g^−1^) were determined based on the CV curves by the formula [32].

(3) $$C_{g}=\frac{\int i\left(V\right)\mathrm{dV}}{2\mathrm{mv}\Delta V}$$

The following formulas were used to define the specific energy density (E, mWh cm^−3^) and power density (P, W cm^−3^) under the dynamic conditions of cyclic voltammetry [29].

(4) $$E=\frac{1}{2}C_{V}{\left(\Delta V\right)}^{2}\frac{1}{3600}$$

(5) $$P=\frac{E}{\Delta t}\times 3600$$

where I is the applied current (A), i(V) is the response current (A), V (cm^3^) and m (g) are the total volume and the total mass of active electrode materials in the measuring system, ν is the potential scan rate (V s^−1^), ΔV (V) is the voltage range, i.e., 1 V during the charge–discharge process, Δt (s) is the discharging time, respectively, C_V_ (F cm^−3^) and C_g_ (F g^−1^) are the volumetric and gravimetric capacitance of the device, E (mWh cm^−3^) and P (W cm^−3^) are the energy and power densities, respectively.

## 3. Results and Discussions

Following the HTHP method, we have recently successfully fabricated highly ordered graphite using natural lignite, which is regarded as an inferior coal resource that is difficult to graphitize compared with the commonly high carbon content anthracite [27]. The enhanced graphitization degree of lignite under HTHP can be characterized by XRD and Raman, as shown in Figure S1a,b. The XRD of raw lignite has a relatively broad (002) peak because there is a large amount of disordered structures and amorphous carbon in lignite. After the HTHP process, it is noted that the (002) peak of the sample becomes strong and sharp, located at 26.4° (corresponding to a *d*-spacing of ~3.36 Å), and a small (110) peak appears, implying the feature of lignite-based synthetic graphite with high orientation. The Raman spectra of samples after the HTHP process and raw lignite show the G-band and the D-band are around 1590 cm^−1^ and 1350 cm^−1^, respectively [33]. Lignite-based synthetic graphite exhibits a prominent G-band and a weak D-band, and the ratio of D-band and G-band intensity is I_D_/I_G_ = 0.17, further confirming the formation of high-quality graphite. The morphology and nanostructure analysis of lignite after HTHP pyrolysis were investigated by SEM and TEM. It can be seen that after the HTHP process the sample displays a sheet-like structure (Figure S1c). Figure S1d provides a typical low-magnification TEM image of lignite-based synthetic graphite. According to the pattern of the corresponding selected area electron diffraction (SAED), a typical diffraction ring with some bright spots of synthetic graphite is clarified, indicating a partial amorphization structure in the lignite-based synthetic graphite.

**Figure 1:**
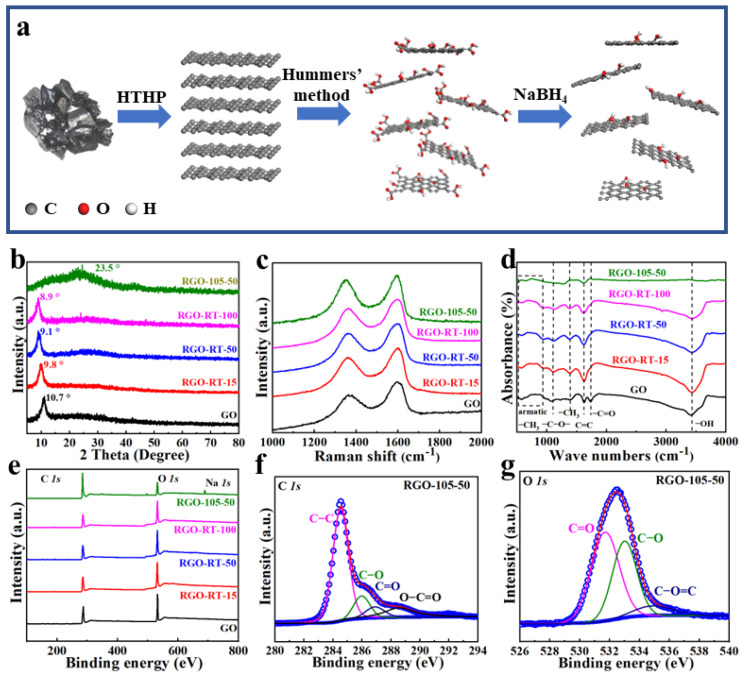
(**a**) Schematic illustration showing the fabrication process of the lignite-derived RGO. (**b**) XRD patterns, (**c**) Raman spectra, (**d**) FTIR spectra, and (**e**) XPS survey spectrum of GO and RGO conducted on different molar concentrations of NaBH_4_ at room temperature and 50 mM NaBH_4_ at 105 °C. (**f**) High-resolution C *1s* XPS spectrum and (**g**) high-resolution O *1s* XPS spectrum of RGO-105-50.

Figure 1a shows a representative preparation procedure of RGO nanomaterials. Firstly, high-quality graphite was prepared by HTHP. Then the Hummers method was successfully utilized to obtain GO. Successively, the RGO was converted by chemical reduction using various molar concentrations of NaBH_4_ and different treatment temperatures to improve its reducing capacity [34]. Systematic characterization analyses including XRD, Raman, FTIR, and XRS were used to investigate the effects of molar concentrations and reduction temperatures on the degree of reduction. The XRD patterns of GO, RGO-RT-15, RGO-RT-50, RGO-RT-100, and RGO-105-50 are shown in Figure 1b. The calculated interlayer distance from the (002) peak of GO is 8.27 Å (2θ = 10.7°), which is larger than the value for synthetic graphite (3.38 Å) [35]. The generation of oxygen-containing functional groups (hydroxyl, epoxy, and carboxyl) in the interlamellar gap of graphite is presumed to be the reason for the expanded interlayer distance. Under the reduction of NaBH_4_, the interlayer distance of RGO-RT-15 was further expanded to 8.9 Å (2θ = 9.8°). With further improving the molar concentration of the reducing agent, the *d*-spacing of RGO was slightly expanded from 9.6 Å (2θ = 9.1°) to 9.8 Å (2θ = 8.9°) for RGO-RT-50 and RGO-RT-100. When GO was reduced by 50 mM of reductant at 105 °C, the steep peak of RGO-105-50 vanished entirely, and a broad peak near 3.7 Å (2θ = 23.5°) occurred instead. This implies that high reduction temperature is more favorable for promoting deoxygenation of GO even under medium concentration of reducing agent.

Raman spectroscopy was adopted to explore the structural evolution under different reducing conditions. All of the spectra have significant D-band and G-band (Figure 1c). The peak position of the G band for GO and RGO samples basically remains unchanged, while the peak of the D band is red-shifted as the degree of reduction increases. Moreover, the ratio of the intensities of the D and G bands (I_D_/I_G_) in the Raman spectra can be used to monitor the formation of defects [36]. As depicted in Figure S2, the I_D_/I_G_ ratio increases significantly even at low reductant concentrations in comparison with GO, whereas only a slight increase is observed in the samples of RGO-105-50, RGO-RT-100, and RGO-105-50. A higher I_D_/I_G_ ratio can be assigned to more structural defects or partially disordered structure in the graphene sheets. This observation shows the removal of oxygen atoms would promote the conversion of sp^3^ bonds to sp^2^ bonds and introduce more defects during the reduction process [37]. The reduction evolution of GO by NaBH_4_ was also recorded by FTIR spectroscopy analysis (Figure 1d). For the GO sample, the FTIR spectrum of GO shows a prominent peak at 3435 cm^−1^, which resulted from the presence of the O–H stretching of intercalated water. The peaks at about 1735 cm^−1^ and 1628 cm^−1^ are assigned to carboxyl/carbonyl (C=O) and aromatic (C=C) stretching groups, respectively [38]. The peak that occurs at 1380 cm^−1^ is associated with the –CH_3_ group, while the peak that occurs at 1104 cm^−1^ stems from –C–O– stretching. The deformation vibration of the –CH outside the aromatic ring is responsible for the low-intensity absorption bands that can be seen between 900 and 700 cm^−1^. When GO is reduced by NaBH_4_ at RT, it is clearly seen that the C=O and C–O species do not disappear, demonstrating that the reduction process is not completed. When the reduced temperature up to 105 °C, there are fewer peaks observed in RGO-105-50, suggesting an efficient removal of oxygen-containing functional groups.

The chemical states of different elements and the presence of functional groups of GO and RGO samples were probed by XPS. Carbon and oxygen are the most prominent elements in the survey scan spectra of all samples (Figure 1e), while there is a Na *1s* peak in sample RGO-105-50, which is derived from the residual NaBH_4_. The high resolution C *1s* region spectra of RGO-105-50 demonstrates four types of carbon bonds at binding energies of 284.5, 286.1, 286.7, and 288.4 eV, corresponding to C–C, C–O, C=O, and O–C=O, respectively [39]. There are the same types of carbon bonds in GO, RGO-RT-15, RGO-RT-50, and RGO-RT-100 (Figure S3a–d). It can be found that the peak intensity of C–O bonds displays a remarkable decrease contrasted with that of C–C as the molar concentrations of NaBH_4_ increase. When the reduced temperature is 105 °C, C–O group is effectively removed even at the mid-range molar concentration of NaBH_4_. Table S1 lists the areas of the contributing peaks. During chemical reduction, it is of particular note that NaBH_4_ can effectively reduce C–O, while having a moderate capability in reduction of C=O, and is basically invalid for the reduction of O–C=O groups. Figure 1g and Figure S3e–h show the fitted O *1s* region of GO, RGO-RT-15, RGO-RT-50, RGO-RT-100, and RGO-105-50. The three O *1s* peaks centering at 531.7 eV (C=O), 532.9 eV (C–O), and 534.9 eV (O–C=O) are found, and the changes in the intensity of these peaks are in accordance with what has been observed in C *1s* spectra. The C:O atomic ratios of GO, RGO-RT-15, RGO-RT-50, RGO-RT-100, and RGO-105-50, obtained by XPS analysis, are 1.95, 1.65, 1.65, 1.59, and 4.04. When GO was reduced at room temperature, the C:O ratio decreased slightly as the NaBH_4_ concentration increased. However, C:O atomic ratio of RGO-105-50 increased dramatically to 4.04, confirming reduction temperature is more conducive to the reduction effect [40].

**Figure 2:**
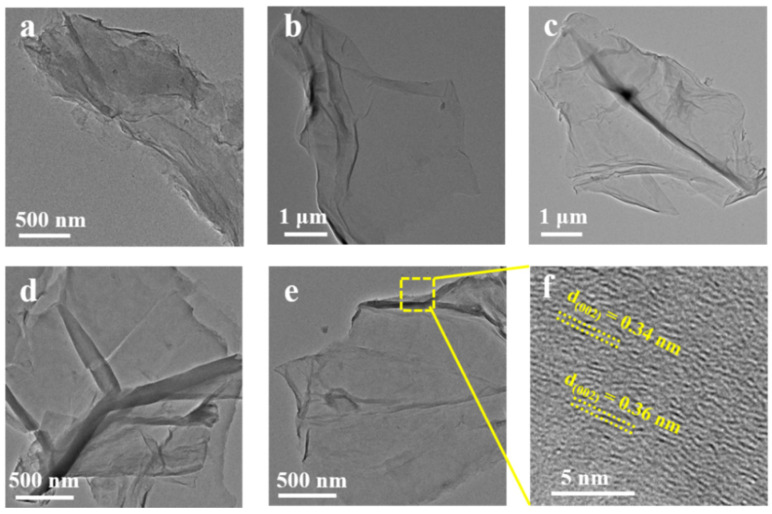
TEM micrographs of (**a**) GO, (**b**) RGO-RT-15, (**c**) RGO-RT-50, (**d**) RGO-RT-100, and (**e**) RGO-105-50. HRTEM micrographs of (**f**) RGO-105-50.

The morphology and structure of the GO and RGO nanosheets were further investigated by TEM. Figure 2 depicts the TEM images of GO, RGO-RT-15, RGO-RT-50, RGO-RT-100, and RGO-105-50, all of these displaying half-transparent sheet-like structure. When lignite was pretreated by HTHP, the size of lignite-derived GO was found to be remarkably increased to 2.8 μm larger than that of GO (size: 40–200 nm) obtained by leaching low-grade coal with HNO_3_ [26]. As with the enhanced reduction degree, the RGO show the less obvious folding features, affirming the removement of oxygen functional groups in agreement with the FTIR and XPS results. Using the HRTEM image (Figure 2d), the inter-planar distance is calculated to be between 0.34 and 0.36 nm, which is close to the value of 0.337 nm for graphite [35].

**Figure 3:**
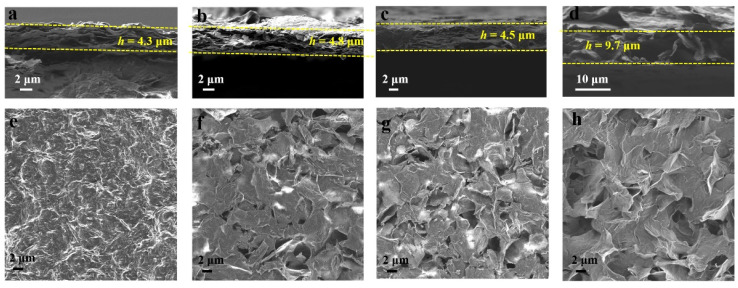
SEM images showing the cross-sectional view of (**a**) RGO-RT-15, (**b**) RGO-RT-50, (**c**) RGO-RT-100, and (**d**) RGO-105-50. SEM images showing the top view of (**e**) RGO-RT-15, (**f**) RGO-RT-50, (**g**) RGO-RT-100, and (**h**) RGO-105-50.

The detailed morphologies of the as-fabricated GO and RGO films were characterized by SEM. As seen by the cross-sectional view of GO, RGO-RT-15, RGO-RT-50, RGO-RT-100, RGO-105-50, and c-RGO-105-50 (Figure 3a–d and Figure S3a,b), the film height was measured to 4.1, 4.3, 4.8, 4.5, 9.7, and 10.4 μm, respectively. There are nano carbon structures in the GO, RGO-RT-15, RGO-RT-50, RGO-RT-100 films, and all these electrodes are agglomerated into densely compact structure, while for RGO-105-50 and c-RGO-105-50, the RGO sheets messily stack on each other, displaying loose structure, which is useful for increasing the ion accessible area and thus improving electrochemical energy storage performance. From the top view of SEM images (Figure 3e–h and Figure S3c,d), RGO-105-50 and c-RGO-105-50 display abundant wrinkles and numerous voids in comparison with GO, RGO-RT-15, RGO-RT-50, and RGO-RT-100.

The electrochemical energy storage performance of the RGO materials were firstly evaluated by CV and GCD measurements using the 3-electrode system (Figures S5a,b, S6a,b, S7a,b, and S8a,b). As we can see, the CV curves of the RGO materials possess the characteristic of quasi-rectangular nature. In addition, the GCD plots exhibit quasi-linear nature which is in the agreement with the CV results. Both the CV and GCD curves indicate the RGO-105-50 electrode has the highest specific capacitance. Specifically, the gravimetric capacitance of the RGO-105-50 is 30.1 F g^−1^ at 0.05 A g^−1^, superior to RGO-RT-15 (1.1 F g^−1^), RGO-RT-50 (1.5 F g^−1^), and RGO-RT-100 (5.9 F g^−1^), as shown in Figures S5c, S6c, S7c, and S8c. The Nyquist plot of RGO-105-50 exhibits an even steeper slope of the linear plots in the low-frequency regions, suggesting the high reduction degree is beneficial to facilitate ion diffusion in energy storage process (Figures S5d, S6d, S7d, and S8d).

**Figure 4:**
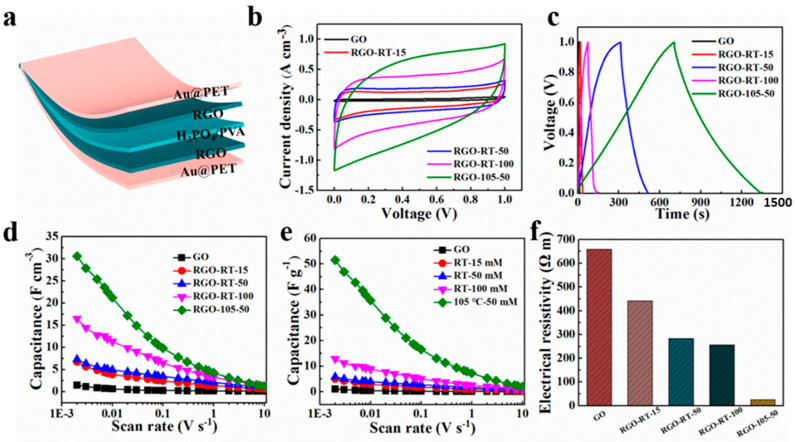
(**a**) The schematic of the structure of flexible supercapacitor. (**b**) CV curves and (**c**) GCD curves of GO, RGO-RT-15, RGO-RT-50, RGO-RT-100, and RGO-105-50 measured at a scan rate of 0.05 V s^−1^ and current density of 0.05 A cm^−3^, respectively. Volumetric (**d**) and mass capacitance (**e**) of GO, RGO-RT-15, RGO-RT-50, RGO-RT-100, and RGO-105-50 as a function of the scan rate from 2 mV s^−1^ to 10 V s^−1^. (**f**) The electrical resistivity of GO, RGO-RT-15 RGO-RT-50, RGO-RT-100, and RGO-105-50 measured via a four-point probe method.

The typical electrochemical properties of the lignite-derived GO and RGO materials were also measured in two-electrode cell configurations using PVA/H_3_PO_4_ electrolyte. A schematic diagram of flexible supercapacitor is depicted in Figure 4a, containing electrode film, H_3_PO_4_/PVA gel electrolyte, and Au-coated PET substrate. The cyclic voltammetry method was used to evaluate the electrochemical performance of the lignite-derived GO and RGO supercapacitors at different scan rates ranging from 2 mV s^−1^ to 10 V s^−1^ (Figures S9a–d, S10a–d, S11a–d, S12a–d, and S13a–d). It can be seen that, with the exception of the CV curves of GO at low scan rates of less than 0.01 V s^−1^, other CV curves exhibit nearly ideal rectangular profile. The deviation from a rectangular shape is most likely caused by the irreversible reaction of oxygen functional groups on graphene with residual water in the electrolyte. As shown in Figures S9e, S10e, S11e, S12e, and S13e, the energy storage performance of GO, RGO-RT-15, RGO-RT-50, RGO-RT-100, and RGO-105-50 were further measured by GCD. The curves of RGO-105-50 show more like symmetric linear shape than other lignite-derived RGO supercapacitors, indicating a good capacitive performance. Meanwhile, the electrochemical performance of the c-RGO-105-50 electrode was also conducted under the same test condition (Figure S14a–e). Figure 4b shows the CV curves of GO, RGO-RT-15, RGO-RT-50, RGO-RT-100, and RGO-105-50 acquired at a scan rate of 0.05 V s^−1^. Obviously, the current density of RGO-105-50 is higher than those of GO and other RGO reduced with NaBH_4_ at RT, implying improved electrochemical performance due to efficient reduction degree. Besides, the discharge time of RGO-105-50 is longer than those of other four kinds of electrodes (Figure 4c).

The specific capacitance values of the lignite-derived GO and RGO electrodes obtained from the CV curves at different scan rates are presented in Figure 4d. The RGO-105-50 electrode has the highest specific capacitance (30.6 F cm^−3^) at 2 mV s^−1^, which is superior to GO (1.5 F cm^−3^), RGO-RT-15 (6.7 F cm^−3^), RGO-RT-50 (7.3 F cm^−3^), and RGO-RT-100 (16.5 F cm^−3^). Besides, the RGO-105-50 electrode maintains maximum capacitance in the entire measurement region, while the difference in specific capacitance of these supercapacitors becomes inconspicuous as the scan rate increasing to 10 V s^−1^. Excitingly, the storage capacitance of RGO-105-50 is slightly better than that of c-RGO-105-50 (28.02 F cm^−3^ at 2 mV s^−1^ as shown in Figure S14f). Furthermore, the gravimetric capacitance of the RGO-105-50 is 51.5 F g^−1^ better than that of the GO and other RGO supercapacitors. The electrical resistivity of GO, RGO-RT-15, RGO-RT-50, RGO-RT-100, and RGO-105-50 films were measured by a four-point probe method. The electrical resistivity of RGO-105-50 possesses the minimum value, which is one of the reasons that RGO-105-50 has the best energy storage performance.

**Figure 5:**
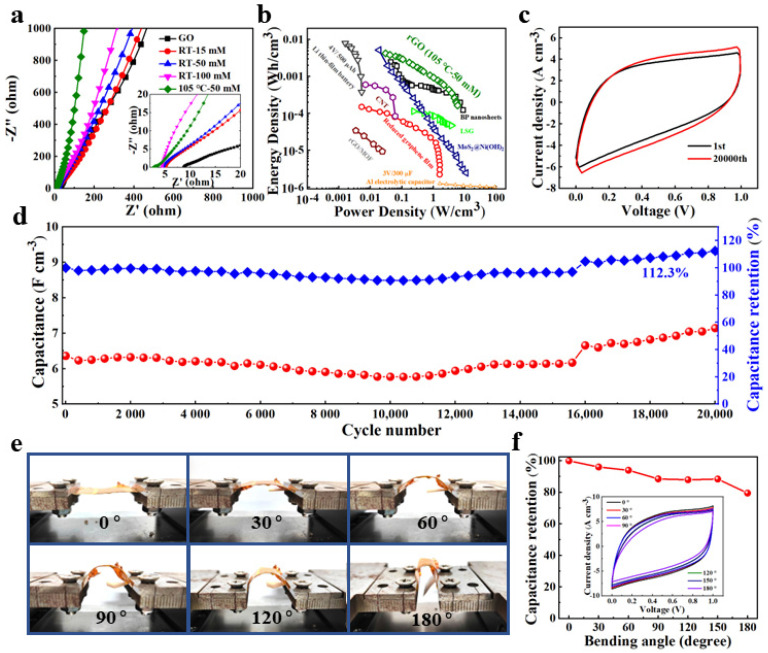
(**a**) Nyquist plots of GO, RGO-RT-15 RGO-RT-50, RGO-RT-100, and RGO-105-50. (**b**) Ragone plot of RGO-105-50 in comparison with commercial energy storage systems and reported supercapacitors. (**c**) Comparison of CV curves of RGO-105-50 supercapacitor at the 1st cycle and 20,000th cycle. (**d**) Cyclic stability of RGO-105-50 supercapacitor over 20,000 cycles at 0.5 V s^−1^. (**e**) Digital photographs of RGO-105-50 supercapacitor at different bending states. (**f**) The capacitance retention of RGO-105-50 supercapacitor measured at 0.5 V s^−1^. The inset shows the basically coincident CV profile of RGO-105-50 supercapacitor.

To further investigate the origin of the excellent energy storage performance, EIS is used to evaluate the electron/ion transport kinetics of electrodes [41]. Figure 5a depicts the obtained Nyquist plots of the GO and RGO materials at frequencies ranging from 10^5^ to 10^−2^ Hz. In the low-frequency zone, the straight-line part of the RGO-105-50 is more parallel to the imaginary axis than that of GO and other RGO materials, indicating that this electrode has a higher mobility of electron and ion. In addition, the lack of a semicircle at the high-frequency zone of the RGO-105-50 electrode suggests a low charge transfer resistance, which is beneficial for the rapid formation of an electrical double layer in the electrode. The equivalent series resistance (ESR), which includes the resistance of the electrolyte, intrinsic resistance of substrate, and contact resistance, can be obtained by the intersection of the curve with the real axis at high frequency. According to the inset of Figure 5a, the ESR values for GO, RGO-RT-15, RGO-RT-50, RGO-RT-100, and RGO-105-50 are 8.7 Ω, 5.2 Ω, 4.7 Ω, 4.4 Ω, and 2.7 Ω, respectively, indicating that the RGO-105-50 material has high electrode conductivity in agreement with the result of electrical resistivity tests (Figure 4f). It can be demonstrated that the RGO-105-50 electrode shows the advantage of ion diffusion during the electrochemical energy storage process, benefiting from the efficient reduction of C=O and C–O groups induced by NaBH_4_.

To further display the practical applications of the as-obtained lignite-derived RGO flexible supercapacitors, the Ragone plot of RGO-105-50 compared with previously reported energy storage devices, such as a lithium thin-film battery (4 V/500 mA h) and an aluminum electrolytic capacitor (3 V/300 mF) [42], is shown in Figure 5b. Note that the RGO-105-50 device could achieve a high energy density of 4.24 mW h cm^−3^ and a power density of 2.89 W cm^−3^, surpassing commonly studied energy storage devices based on two-dimensional and carbon-based materials, such as reduced graphene film by CH_4_ plasma reduction (1.6 W cm^−3^ and 0.15 mWh cm^−3^) [43], laser-scribed graphene (3.8 W cm^−3^ and 0.11 mWh cm^−3^) [44], nanocomposites MoS_2_@Ni(OH)_2_ (11 W cm^−3^ and 5.2 mWh cm^−3^) [45], three-dimensional graphene and metal-organic framework composite (0.02 W cm^−3^ and 0.03 mWh cm^−3^) [46], CNT film (0.01 W cm^−3^ and 0.08 mWh cm^−3^) [47], and black phosphorus nanosheets (8.83 W cm^−3^ and 2.47 mWh cm^−3^) [29]. The comparison of energy storage performance of RGO-105-50 with other electrode materials is shown in Table S2.

One of the most critical properties to be evaluated before putting flexible supercapacitors into practical application is their long-term cycling stability. In this work, the RGO-105-50 device was tested for stability at 0.5 V s^−1^. The CV curve of RGO-105-50 after 20,000 long-term cycles (Figure 5c) possesses a larger integral area than its initial CV curve, confirming its remarkable cycling stability. As depicted in Figure 5d, the retention rate of RGO-105-50 shows slight growth over 20,000 cycles, finally reaching 112.3% of the initial capacitance, superior to that of c-RGO-105-50 fabricated by the same processing technic (101.8% after 20,000 long-term cycles as shown in Figure S14g). The enhancement of the capacity after long cycling is due to the adequate penetration of the electrolyte and the gradual activation of the surface. Furthermore, the CV curves of RGO-105-50 were measured under various bending angles in the range of 0–180° (Figure 5e). Notably, RGO-105-50 flexible supercapacitor subjected to different bending angles show similar CV curves even at extreme degrees of bending (Figure 5f), and the capacitance remains 79.5% of the capacitance of flat state without destroying the structural integrity, illustrating the excellent mechanical flexibility and potential application in flexible energy storage devices.

To further explore the micro-structure and surface chemical compositions of RGO-105-50 after 20,000 cycles, SEM and XPS were adopted. After electrochemical measurements, RGO nanoflakes tend to stack on each other, displaying a smooth surface (Figure S15a). From the XPS spectra of C *1s* and O *1s* in Figure S15b–c, the C–O bond is significantly enhanced in complex electrochemical energy storage processes. The change in relative peak strength of C–O bond is believed to offer a good pseudocapacitance effect, which is responsible for the improvement of the retention rate of RGO-105-5 [48]. In addition, Figure S16 depicts the obtained Nyquist plots of RGO-105-50 after 20,000 cycles, the EIS test of RGO-105-50 after 20,000 cycles shows that the ESR increases to 8.8 Ω and the low frequency straight line is closer to the real axis, indicating the slower ion diffusion process and lower ion transport.

## 4. Conclusions

In summary, we have demonstrated an optimizing route for the mass production of large-size GO derived from low-value lignite, that benefits from the highly ordered graphite material prepared by the HTHP method. Lignite-derived RGO can be obtained by the efficient reduction of NaBH_4_, in which the reduction temperature is more conducive to the degree of reduction compared with molar concentrations of reductive agent. The RGO-105-50 electrode prepared by vacuum filtration features porosity and high conductivity, which improves the contact area between electrode and electrolyte and facilitates electron/ion transport. As is expected, the RGO-105-50 electrode exhibits a high volumetric capacitance of 30.6 F cm^−3^, an excellent volumetric energy density of 4.2 mW h cm^−3^, a power density of 5.9 W cm^−3^, and exceptional flexibility, i.e., 79.5% of the initial specific capacitance even under 180° bending. Remarkably, the specific capacitance of the assembled RGO-105-50 flexible supercapacitors remains about 112.3% after 20,000 cycles, which is higher than that of RGO prepared by the chemical reduction of purchased GO (101.8% retention of the initial capacitance after 20,000 cycles). The current work not only demonstrates an effective route for the synthesis of the large-size RGO sheets in the manner of large quantity and low cost, but also shows that the lignite-derived RGO can be supposed as a promising candidate of high-performance electrode materials for flexible supercapacitors.

## Data Availability

The data presented in this study are available on request from the corresponding author.

## Supplementary Materials

The following supporting information can be downloaded at: https://www.mdpi.com/article/10.3390/nano12172989/s1, Figure S1: Comparing XRD (a) and Raman (b) results of raw coal and graphite preparing by HTHP. SEM (c) and TEM (d) images of graphite preparing by HTHP with inset showing the corresponding SAED pattern; Figure S2: The ratio of the intensities of the D and G bands histogram of GO, RGO-RT-15, RGO-RT-50, RGO-RT-100, and RGO-105-50; Figure S3: High-resolution C *1s* XPS spectrum of (a) GO, (b) RGO-RT-15, (c) RGO-RT-50, and (d) RGO-RT-100. High-resolution O *1s* XPS spectrum of (e) GO, (f) RGO-RT-15, (g) RGO-RT-50, and (h) RGO-RT-100; Figure S4: SEM images showing the cross-sectional view of the (a) GO and (b) c-RGO-105-50. SEM images showing the top view of the (c) GO and (d) c-RGO-105-50; Figure S5: Electrochemical properties of RGO-RT-15 in three-electrode system. (a) Cyclic voltammograms at various scan rates from 0.01 V s^−1^ to 0.1 V s^−1^. (b) Galvanostatic charging/discharging curves obtained at different current densities from 0.05 A g^−1^ to 0.5 A g^−1^. (c) The gravimetric capacitance of RGO-RT-15 as a function of the current density from 0.05 A g^−1^ to 0.5 A g^−1^. (d) Nyquist plots of RGO-RT-15; Figure S6: Electrochemical properties of RGO-RT-50 in three-electrode system. (a) Cyclic voltammograms at various scan rates from 0.01 V s^−1^ to 0.1 V s^−1^. (b) Galvanostatic charging/discharging curves obtained at different current densities from 0.05 A g^−1^ to 0.5 A g^−1^. (c) The gravimetric capacitance of RGO-RT-50 as a function of the current density from 0.05 A g^−1^ to 0.5 A g^−1^. (d) Nyquist plots of RGO-RT-50; Figure S7: Electrochemical properties of RGO-RT-100 in three-electrode system. (a) Cyclic voltammograms at various scan rates from 0.01 V s^−1^ to 0.1 V s^−1^. (b) Galvanostatic charging/discharging curves obtained at different current densities from 0.05 A g^−1^ to 0.5 A g^−1^. (c) The gravimetric capacitance of RGO-RT-100 as a function of the current density from 0.05 A g^−1^ to 0.5 A g^−1^. (d) Nyquist plots of RGO-RT-100; Figure S8: Electrochemical properties of RGO-105-50 in three-electrode system. (a) Cyclic voltammograms at various scan rates from 0.01 V s^−1^ to 0.1 V s^−1^. (b) Galvanostatic charging/discharging curves obtained at different current densities from 0.05 A g^−1^ to 0.5 A g^−1^. (c) The gravimetric capacitance of RGO-105-50 as a function of the current density from 0.05 A g^−1^ to 0.5 A g^−1^. (d) Nyquist plots of RGO- RGO-105-50; Figure S9: Electrochemical properties of GO flexible supercapacitor. (a–d) Cyclic voltammograms at various scan rates from 2 mV s^−1^ to 10 V s^−1^. (e) Galvanostatic charging/discharging curves obtained at different current densities from 0.03 to 0.2 A cm^−3^; Figure S10: Electrochemical properties of RGO-RT-15 flexible supercapacitor. (a–d) Cyclic voltammograms at various scan rates from 2 mV s^−1^ to 10 V s^−1^. (e) Galvanostatic charging/discharging curves obtained at different current densities from 0.03 to 0.2 A cm^−3^; Figure S11: Electrochemical properties of RGO-RT-50 flexible supercapacitor. (a–d) Cyclic voltammograms at various scan rates from 2 mV s^−1^ to 10 V s^−1^. (e) Galvanostatic charging/discharging curves obtained at different current densities from 0.03 to 0.2 A cm^−3^; Figure S12: Electrochemical properties of RGO-RT-100 flexible supercapacitor. (a–d) Cyclic voltammograms at various scan rates from 2 mV s^−1^ to 10 V s^−1^. (e) Galvanostatic charging/discharging curves obtained at different current densities from 0.03 to 0.2 A cm^−3^; Figure S13: Electrochemical properties of RGO-105-50 flexible supercapacitor. (a–d) Cyclic voltammograms at various scan rates from 2 mV s^−1^ to 10 V s^−1^. (e) Galvanostatic charging/discharging curves obtained at different current densities from 0.03 to 0.2 A cm^−3^; Figure S14: Electrochemical properties of c-RGO-105-50 flexible supercapacitor. (a–d) Cyclic voltammograms at various scan rates from 2 mV s^−1^ to 10 V s^−1^. (e) Galvanostatic charging/discharging curves obtained at different current densities from 0.03 to 0.2 A cm^−3^. (f) Volumetric capacitance of c-RGO-105-50 as a function of the scan rate from 2 mV s^−1^ to 10 V s^−1^. (g) Cyclic stability of c-RGO-105-50 flexible supercapacitor over 20,000 cycles at 0.5 V s^−1^; Figure S15: (a) SEM image of RGO-105-50 after 20,000 cycles. (b) XPS survey spectrum, (c) high-resolution C *1s* XPS spectrum and (d) high-resolution O 1s XPS spectrum of RGO-105-50 after 20,000 cycles; Figure S16: Nyquist plots of RGO-105-50 after 20,000 cycles; Table S1: The elemental composition, the relative atomic percentage of various functional groups of GO, RGO-RT-15, RGO-RT-50, RGO-RT-100, and RGO-105-50; Table S2: The comparison of energy storage performance with different electrode materials.
