# Supplementary material for: Reduced Graphene Oxide Derived from Low-Grade Coal for High-Performance Flexible Supercapacitors with Ultrahigh Cyclability
Source: Nanomaterials (Basel). 2022 Aug 29;12(17):2989. doi: 10.3390/nano12172989 (PMC9457952; doi:10.3390/nano12172989)
Supplement: Supplementary file 1 [file nanomaterials-12-02989-s001.zip › nanomaterials-1865114-supplementary.pdf]

Supplementary Materials

Reduced Graphene Oxide Derived from Low-Grade Coal for High-Performance Flexible Supercapacitors with Ultrahigh Cyclability

Yi-Ming Wang and Chun-Hua Zhang *

College of Safety Science and Engineering, Liaoning Technical University, Fuxin 123000, China
* Correspondence: zhangchunhua@lntu.edu.cn

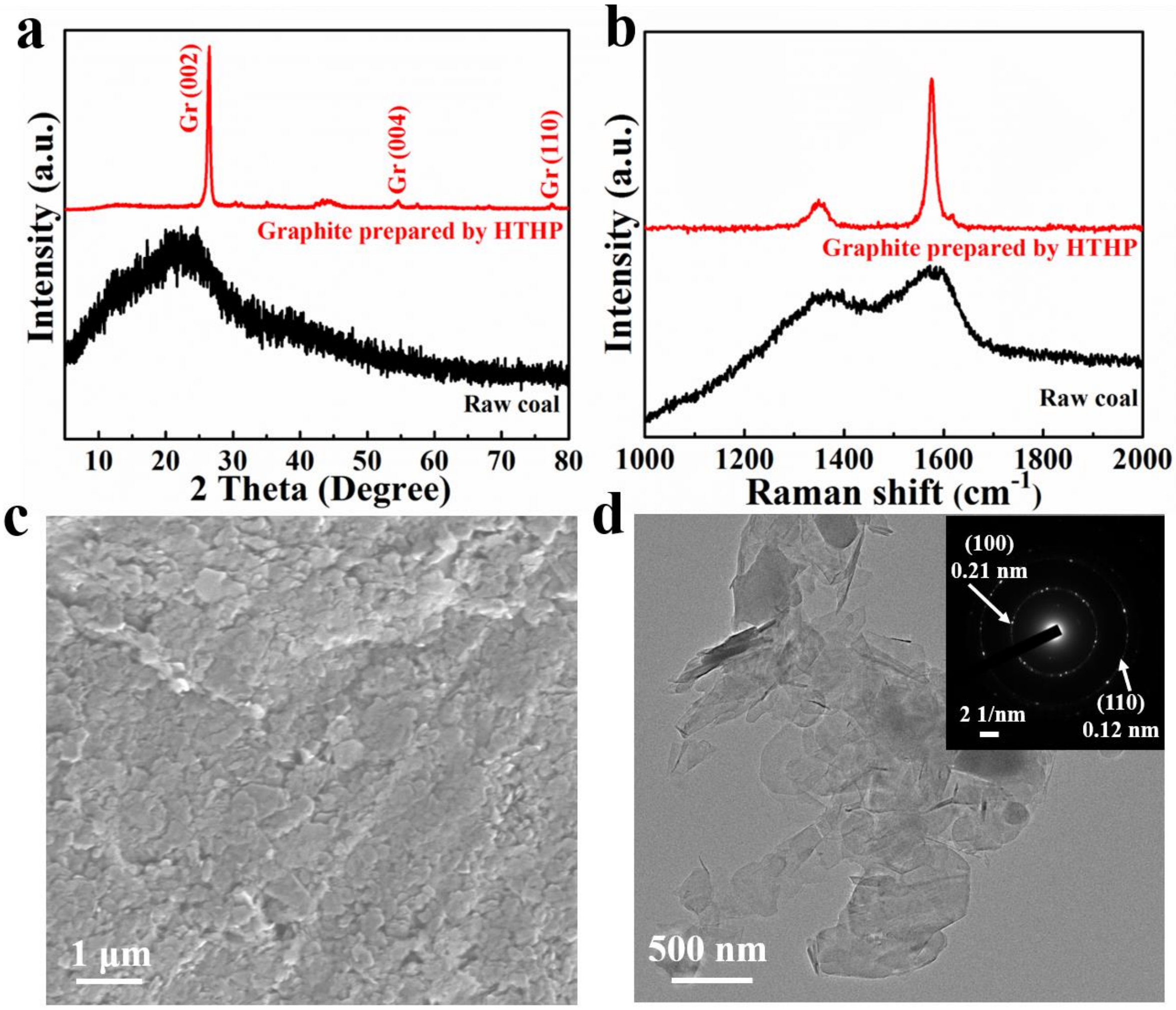

Figure S1. Comparing XRD (a) and Raman (b) results of raw coal and graphite preparing by HTHP. SEM (c) and TEM (d) images of graphite preparing by HTHP with inset showing the corresponding SAED pattern.

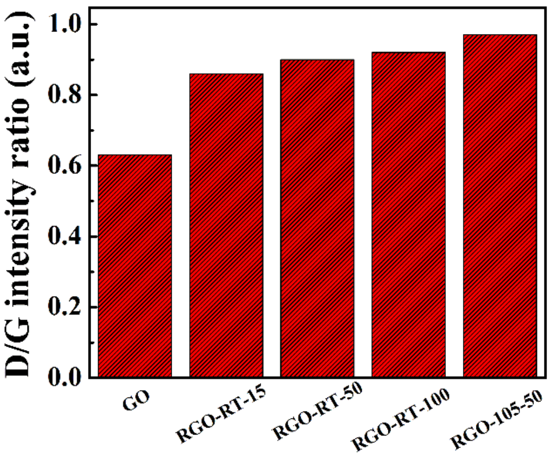

Figure S2. The ratio of the intensities of the D and G bands histogram of GO, RGO-RT-15, RGO-RT-50, RGO-RT-100, and RGO-105-50.

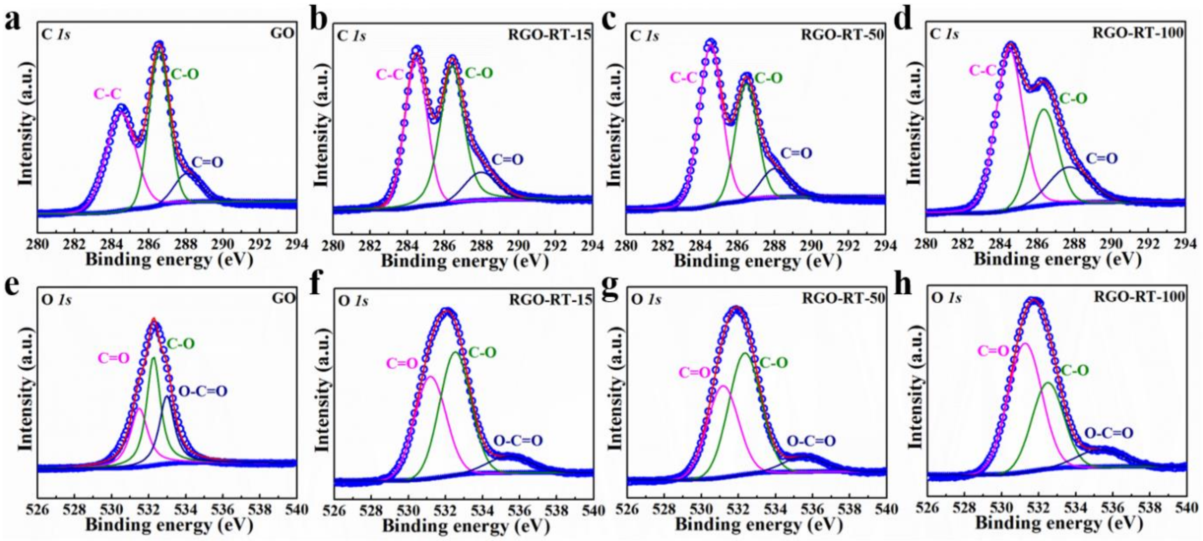

Figure S3. High-resolution C *1s* XPS spectrum of (a) GO, (b) RGO-RT-15, (c) RGO-RT-50, and (d) RGO-RT-100. High-resolution O *1s* XPS spectrum of (e) GO, (f) RGO-RT-15, (g) RGO-RT-50, and (h) RGO-RT-100.

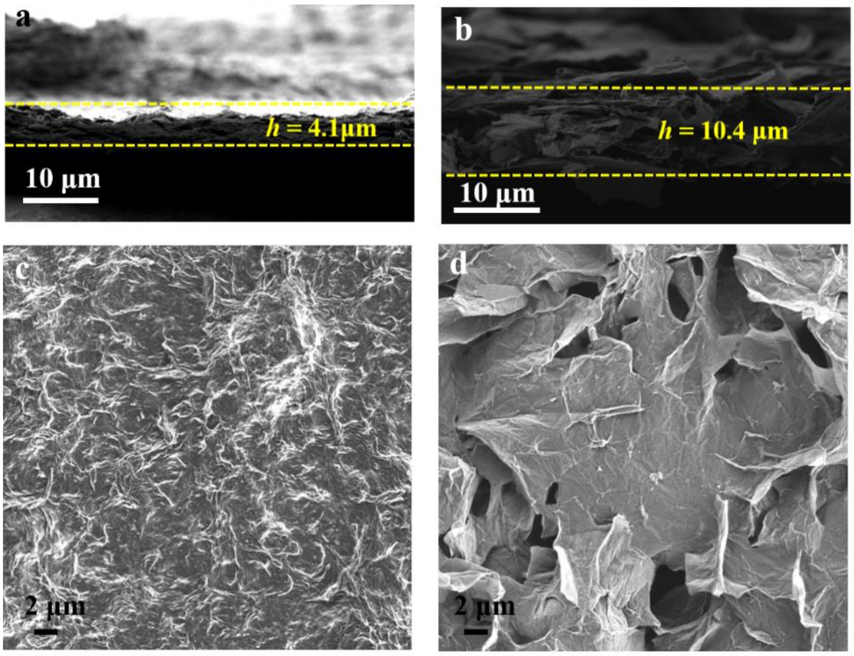

Figure S4. SEM images showing the cross-sectional view of the (a) GO and (b) c-RGO-105-50. SEM images showing the top view of the (c) GO and (d) c-RGO-105-50.

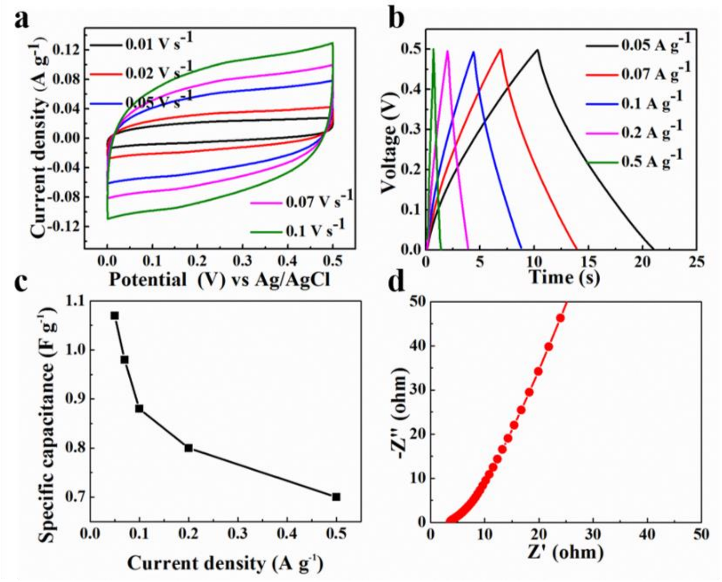

Figure S5. Electrochemical properties of RGO-RT-15 in three-electrode system. (a) Cyclic voltammograms at various scan rates from 0.01 V s^{-1} to 0.1 V s^{-1}. (b) Galvanostatic charging/discharging curves obtained at different current densities from 0.05 A g^{-1} to 0.5 A g^{-1}. (c) The gravimetric capacitance of RGO-RT-15 as a function of the current density from 0.05 A g^{-1} to 0.5 A g^{-1}. (d) Nyquist plots of RGO-RT-15.

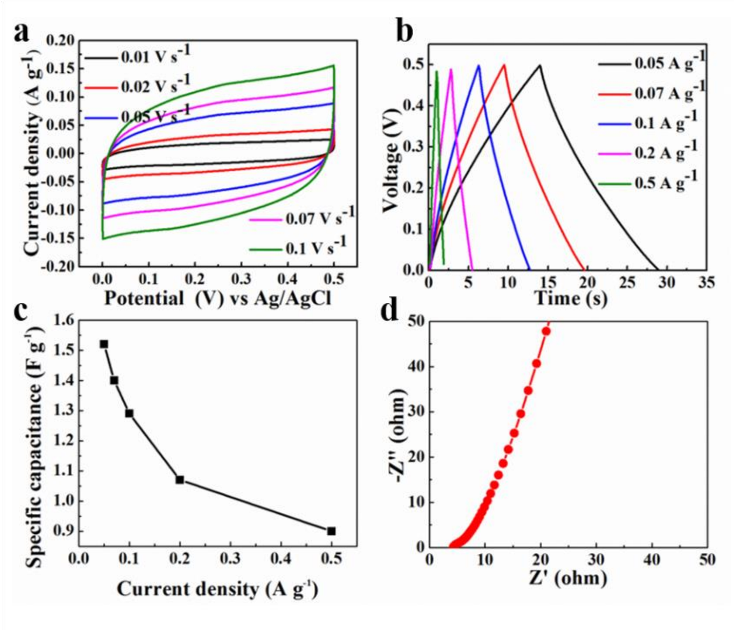

Figure S6. Electrochemical properties of RGO-RT-50 in three-electrode system. (a) Cyclic voltammograms at various scan rates from 0.01 V s^{-1} to 0.1 V s^{-1}. (b) Galvanostatic charging/discharging curves obtained at different current densities from 0.05 A g^{-1} to 0.5 A g^{-1}. (c) The gravimetric capacitance of RGO-RT-50 as a function of the current density from 0.05 A g^{-1} to 0.5 A g^{-1}. (d) Nyquist plots of RGO-RT-50.

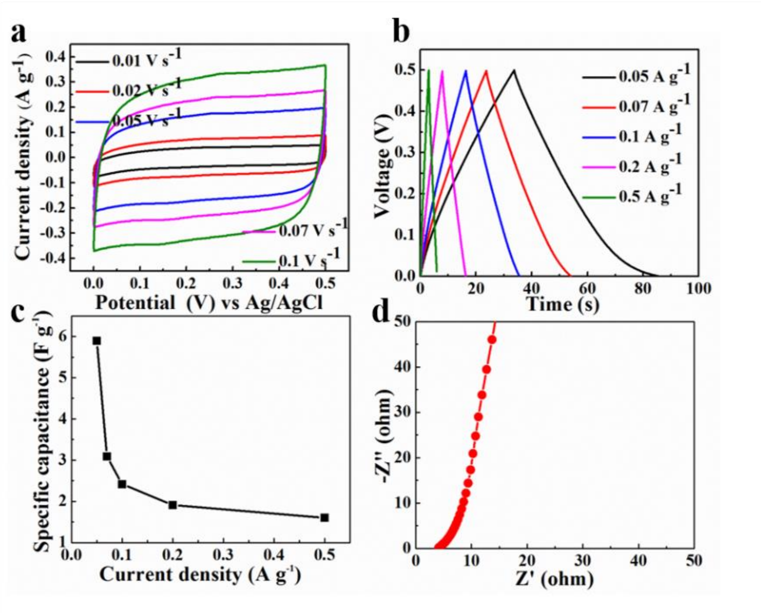

Figure S7. Electrochemical properties of RGO-RT-100 in three-electrode system. (a) Cyclic voltammograms at various scan rates from 0.01 V s^{-1} to 0.1 V s^{-1}. (b) Galvanostatic charging/discharging curves obtained at different current densities from 0.05 A g^{-1} to 0.5 A g^{-1}. (c) The gravimetric capacitance of RGO-RT-100 as a function of the current density from 0.05 A g^{-1} to 0.5 A g^{-1}. (d) Nyquist plots of RGO-RT-100.

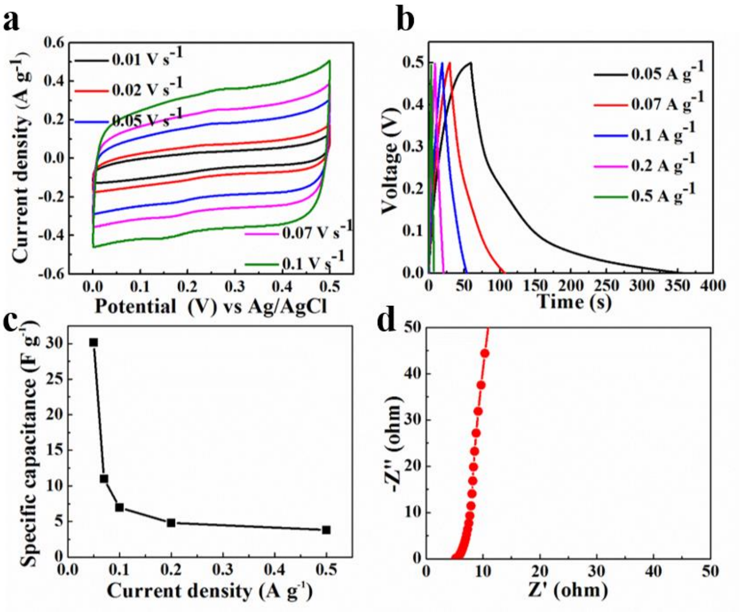

Figure S8. Electrochemical properties of RGO-105-50 in three-electrode system. (a) Cyclic voltammograms at various scan rates from 0.01 V s^{-1} to 0.1 V s^{-1}. (b) Galvanostatic charging/discharging curves obtained at different current densities from 0.05 A g^{-1} to 0.5 A g^{-1}. (c) The gravimetric capacitance of RGO-105-50 as a function of the current density from 0.05 A g^{-1} to 0.5 A g^{-1}. (d) Nyquist plots of RGO- RGO-105-50.

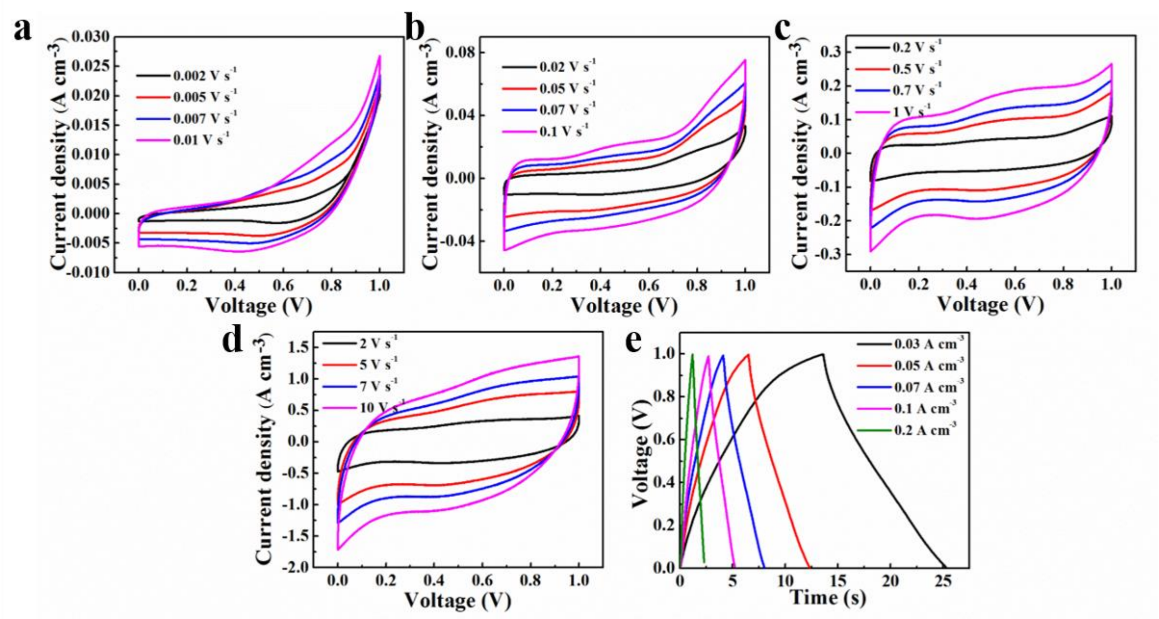

Figure S9. Electrochemical properties of GO flexible supercapacitor. (a-d) Cyclic voltammograms at various scan rates from 2 mV s^{-1} to 10 V s^{-1}. (e) Galvanostatic charging/discharging curves obtained at different current densities from 0.03 to 0.2 A cm^{-3}.

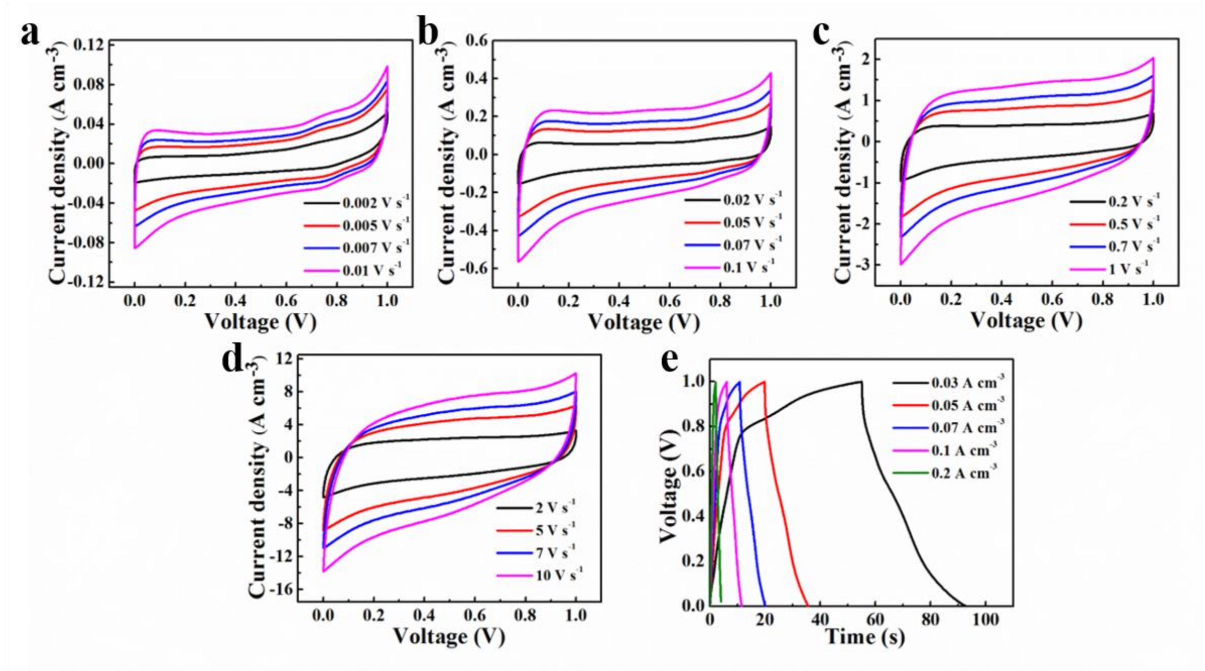

Figure S10. Electrochemical properties of RGO-RT-15 flexible supercapacitor. (a-d) Cyclic voltammograms at various scan rates from 2 mV s^{-1} to 10 V s^{-1}. (e) Galvanostatic charging/discharging curves obtained at different current densities from 0.03 to 0.2 A cm^{-3}.

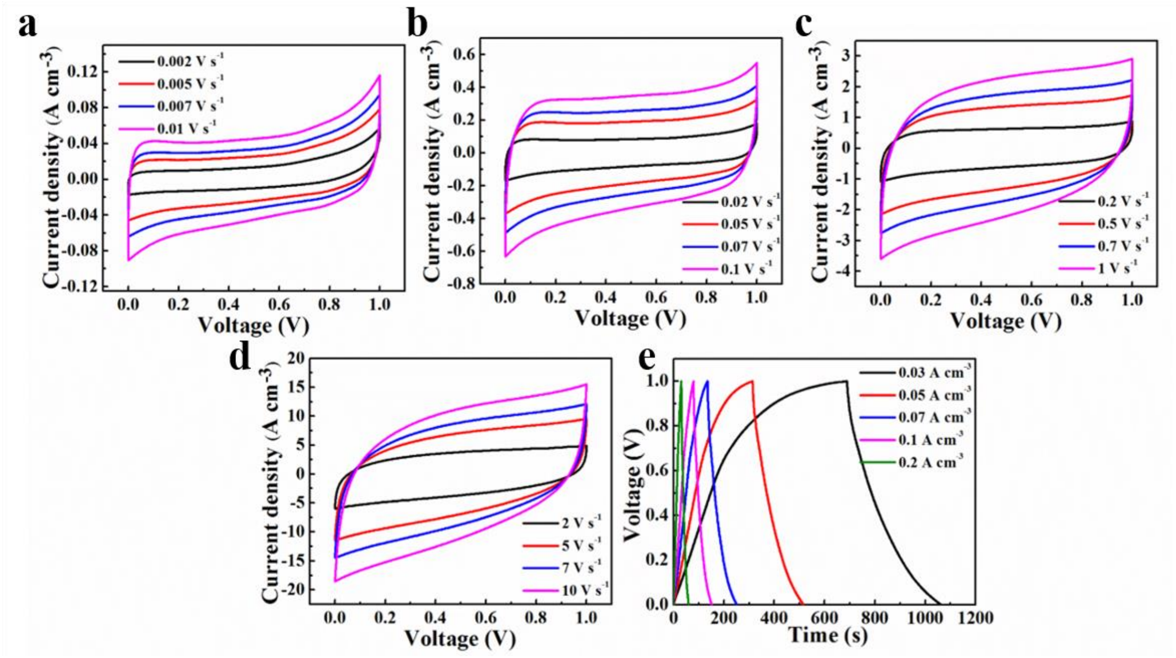

Figure S11. Electrochemical properties of RGO-RT-50 flexible supercapacitor. (a-d) Cyclic voltammograms at various scan rates from 2 mV s^{-1} to 10 V s^{-1}. (e) Galvanostatic charging/discharging curves obtained at different current densities from 0.03 to 0.2 A cm^{-3}.

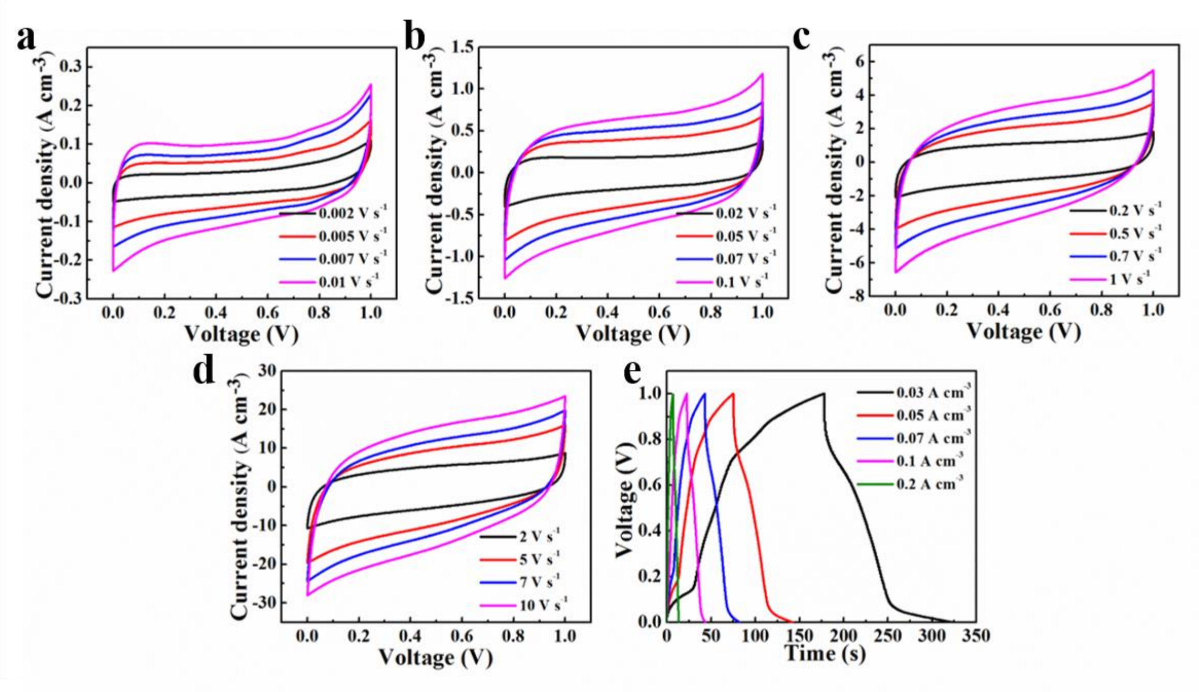

Figure S12. Electrochemical properties of RGO-RT-100 flexible supercapacitor. (a-d) Cyclic voltammograms at various scan rates from 2 mV s^{-1} to 10 V s^{-1}. (e) Galvanostatic charging/discharging curves obtained at different current densities from 0.03 to 0.2 A cm^{-3}.

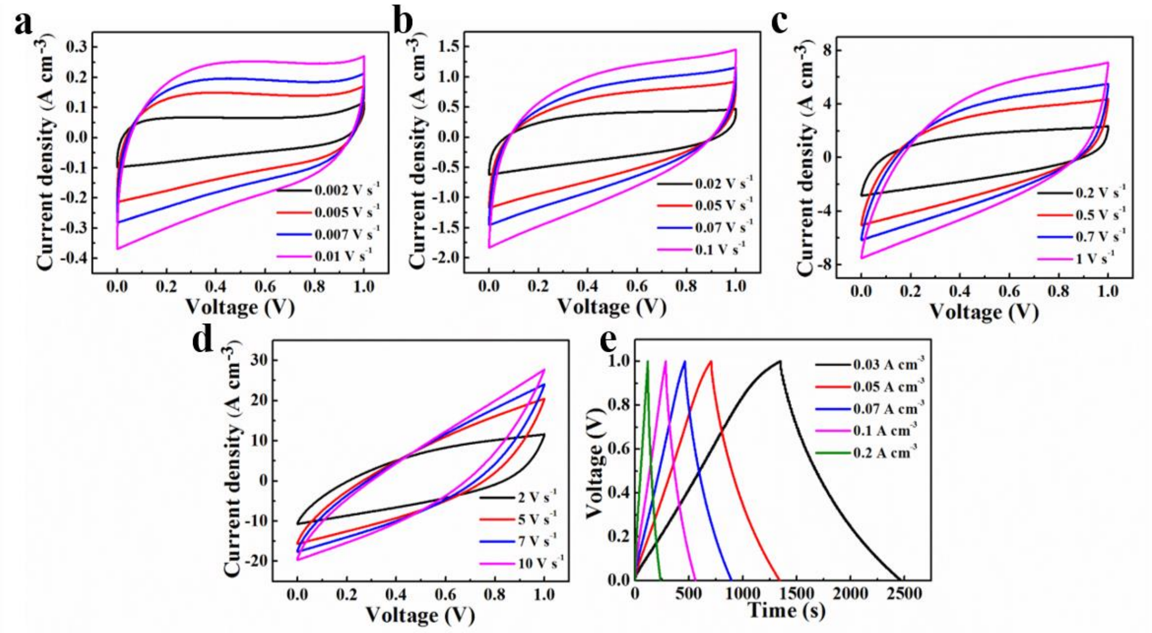

Figure S13. Electrochemical properties of RGO-105-50 flexible supercapacitor. (a-d) Cyclic voltammograms at various scan rates from 2 mV s^{-1} to 10 V s^{-1}. (e) Galvanostatic charging/discharging curves obtained at different current densities from 0.03 to 0.2 A cm^{-3}.

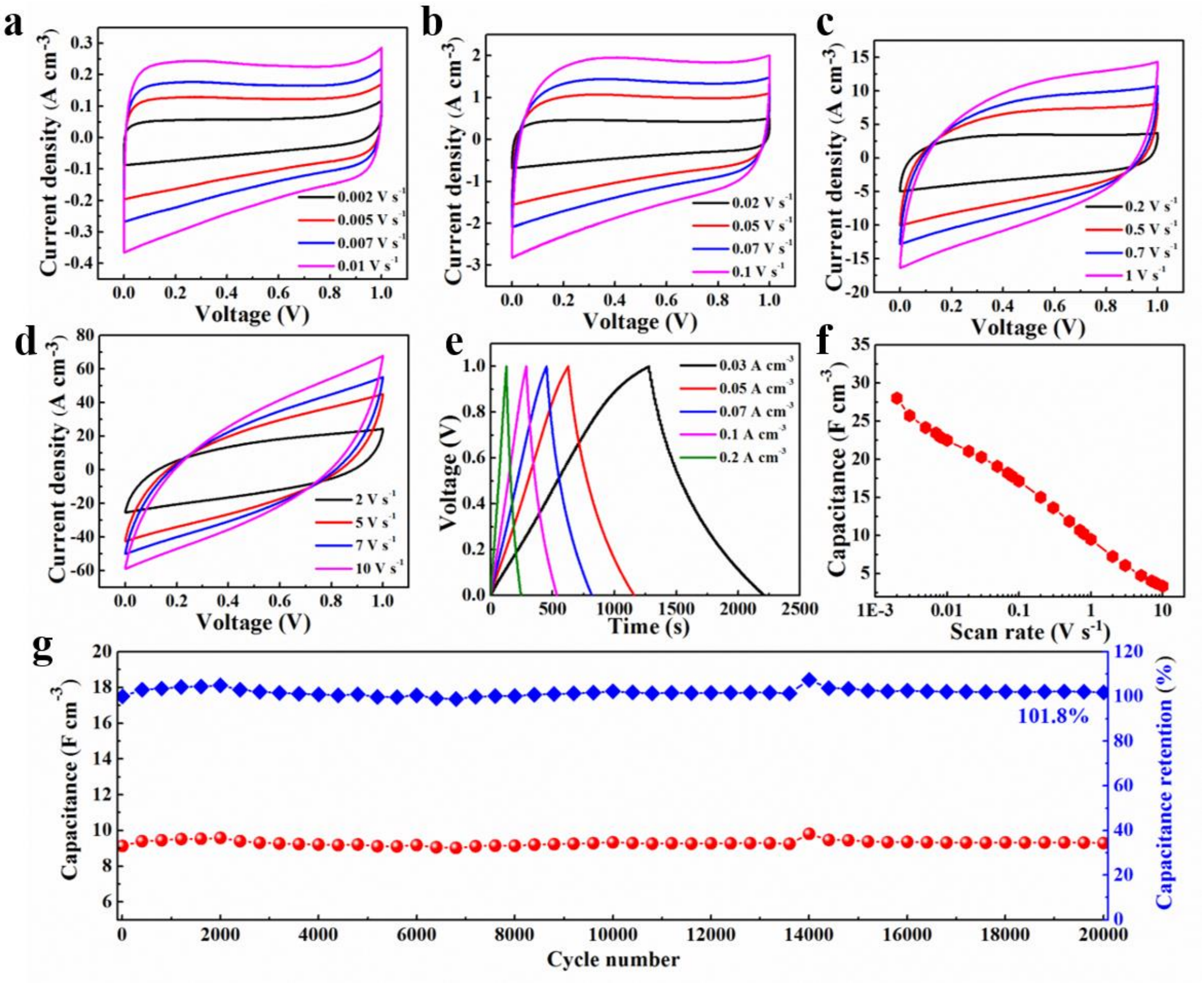

Figure S14. Electrochemical properties of c-RGO-105-50 flexible supercapacitor. (a-d) Cyclic voltammograms at various scan rates from 2 mV s^{-1} to 10 V s^{-1}. (e) Galvanostatic charging/discharging curves obtained at different current densities from 0.03 to 0.2 A cm^{-3}. (f) Volumetric capacitance of c-RGO-105-50 as a function of the scan rate from 2 mV s^{-1} to 10 V s^{-1}. (g) Cyclic stability of c-RGO-105-50 flexible supercapacitor over 20,000 cycles at 0.5 V s^{-1}.

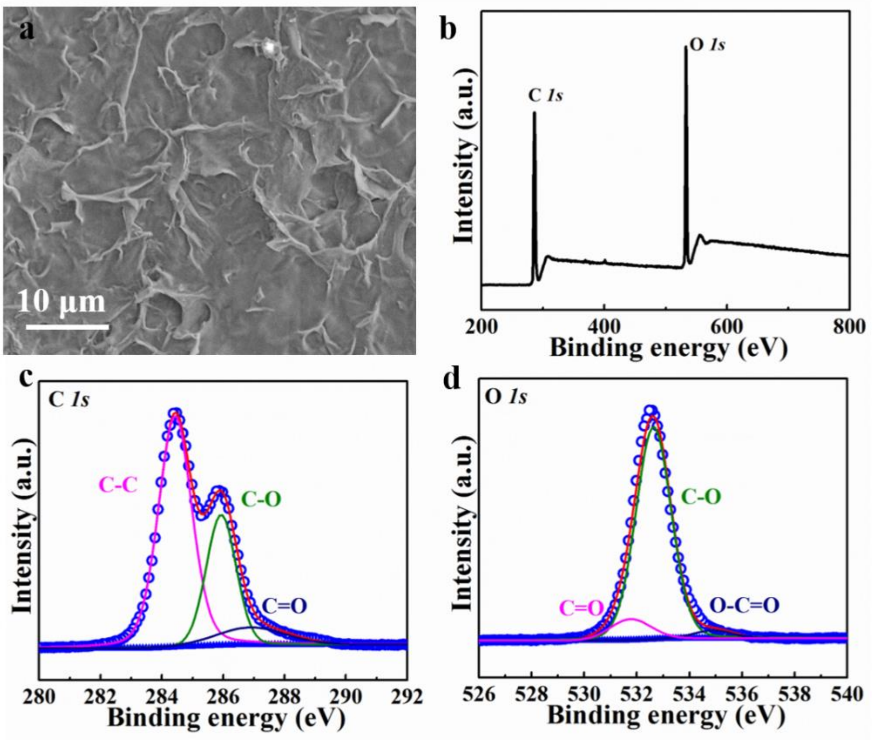

Figure S15. (a) SEM image of RGO-105-50 after 20,000 cycles. (b) XPS survey spectrum, (c) high-resolution C *1s* XPS spectrum and (d) high-resolution O *1s* XPS spectrum of RGO-105-50 after 20,000 cycles.

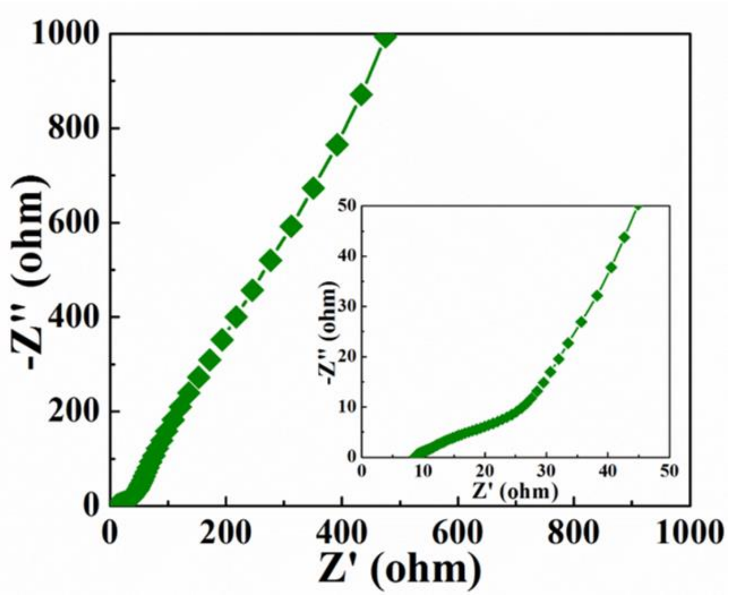

Figure S16. Nyquist plots of RGO-105-50 after 20,000 cycles.

Table S1. The elemental composition, the relative atomic percentage of various functional groups of GO, RGO-RT-15, RGO-RT-50, RGO-RT-100 and RGO-105-50

	C/O ratio	C-C	C-O	C=O	O-C=O
GO	1.95	44.5%	32.1%	13.3%	10.1%
RGO-RT-15	1.65	46.1%	22.1%	17.6%	14.2%
RGO-RT-50	1.65	53.7%	14.9%	19.4%	12.0%
RGO-RT-100	1.59	47.9%	20.4%	18.6%	13.0%
RGO-105-50	4.04	65.1%	13.2%	8.2%	13.5%

Table S2. The comparison of energy storage performance with different electrode materials

Electrode materials	Electrolyte	Volumetric capacitance (F cm^{-3})	gravimetric capacitance (F g^{-1})	energy density (mW h cm^{-3})	power density (W cm^{-3})	Ref.
BP nanosheets	PVA/H_3PO_4	13.75	45.8	2.47	8.83	1
Graphene	PVA/H_2SO_4	17.9		2.5	495	2
Laser-scribed graphene	PVA/H_2SO_4	2.35		0.11	3.78	3
MoS_2@$Ni(OH)_2$	PVA/KOH	37.53	516.4	5.2	11	4
rGO/MOF	PVA/KOH	0.25		0.035	0.072	5
CNT	PVA/H_3PO_4	0.0038	30.3	0.58	0.39	6
RGO-105-50	PVA/H_3PO_4	30.6	51.5	4.24	2.89	This work

1. Hao, C.; Yang, B.; Wen, F.; Xiang, J.; Li, L.; Wang, W.; Zeng, Z.; Xu, B.; Zhao, Z.; Liu, Z.; Tian, Yong. Flexible All-Solid-State Supercapacitors based on Liquid-Exfoliated Black-Phosphorus Nanoflakes. *Adv. Mater.* **2016**, *28*, 3194–3201.
2. Wu, Z.S.; Parvez, K.; Feng, X.; Mullen, K. Graphene-based in-plane micro-supercapacitors with high power and energy densities. *Nat. Commun.* **2013**, *4*, 2487.
3. El-Kady, M.F.; Kaner, R.B. Scalable fabrication of high-power graphene micro-supercapacitors for flexible and on-chip energy storage. *Nat. Commun.* **2013**, *4*, 1475.
4. Hao, C.; Wen, F.; Xiang, J.; Wang, L.; Hou, H.; Su, Z.; Hu, W.; Liu, Z. Controlled Incorporation of $Ni(OH)_2$ Nanoplates Into Flowerlike MoS_2 Nanosheets for Flexible All-Solid-State Supercapacitors. *Adv. Funct. Mater.* **2014**, *24*, 6700–6707.
5. Xu, X.; Shi, W.; Li, P.; Ye, S.; Ye, C.; Ye, H.; Lu, T.; Zheng, A.; Zhu, J.; Xu, L.; et al. Facile Fabrication of Three-Dimensional Graphene and Metal-Organic Framework Composites and Their Derivatives for Flexible All-Solid-State Supercapacitors. *Chem. Mater.* **2017**, *29*, 6058–6065.
6. Song, L.; Cao, X.; Li, L.; Wang, Q.; Ye, H.; Gu, L.; Mao, C.; Song, J.; Zhang, S.; Niu, H. General Method for Large-Area Films of Carbon Nanomaterials and Application of a Self-Assembled Carbon Nanotube Film as a High-Performance Electrode Material for an All-Solid-State Supercapacitor. *Adv. Funct. Mater.* **2017**, *27*, 1700474.
